# Utilization of Long-Acting Contraceptive Methods and Associated Factors among Female Health Care Providers in East Gojjam Zone, Northwest Ethiopia, in 2018

**DOI:** 10.1155/2019/5850629

**Published:** 2019-11-03

**Authors:** Liknaw Bewket Zeleke, Manaye Meku Gella, Hunegnaw Almaw Derseh, Addisu Alehegn Alemu, Eskeziaw Abebe Kassahun, Kelemu Abebe Gelaw

**Affiliations:** ^1^College of Health Sciences, Debre Markos University, Debre Markos, Ethiopia; ^2^Debremarkos Town Health Office, Bahir Dar, Ethiopia; ^3^College of Health Sciences and Medicine, Bahir Dar University, Bahir Dar, Ethiopia; ^4^Faculty of Health Sciences, Woldia University, Woldia, Ethiopia; ^5^College of Health Sciences and Medicine, Wolaita Sodo University, Wolaita Sodo, Ethiopia

## Abstract

**Introduction:**

Sub-Saharan Africa, including Ethiopia, faces serious population and reproductive health challenges, indicated by a higher unmet need for family planning, especially for long-acting contraceptive methods, higher fertility, and population growth rates. The utilization of long-acting reversible contraceptive methods in Ethiopia and in particular in the study area is low.

**Objective:**

This study aimed to assess the utilization of long-acting reversible contraceptive methods among female health care workers in the reproductive age group in East Gojjam Zone, Northwest Ethiopia, in 2018.

**Methods:**

Institutional-based cross-sectional study was conducted from 1 to 30 March 2018. A total of 392 female health care workers have participated. Data were collected by a structured, pretested, and self-administered questionnaire, then entered into Epi-info Version 7, and analyzed by SPSS Version 21. Bivariable and multivariate binary logistic regression analyses were carried out. *p* value <0.05 was considered to declare statistically significant variables.

**Result:**

The current utilization of long-acting contraceptive methods among female health workers was found to be 22.7%. Supportive attitude of their husbands/partners (AOR at 95% CI 4.62 (1.52–14.09)), having <5000 EBrr monthly family income (AOR at 95% CI 2.813 (1.04–7.57)), supportive attitude towards the utilization of long-acting contraceptive methods (AOR at 95% CI 5.13 (2.03–12.95)), and the desire to have 0–2 children (AOR at 95% CI 5.34 (1.80–15.80)) were positively associated factors towards the utilization of long-acting contraceptive methods.

**Conclusion:**

The current utilization of long-acting contraceptive methods was found low. Husbands/partners' supportive attitude, the number of children they want to have, attitude, and monthly family income were identified as significant factors. The East Gojjam Zonal Health Department and other stakeholders should work on the promotion of partners/husbands' involvement in the utilization of long-acting contraceptive methods among reproductive age women, including health care workers.

## 1. Introduction

Family planning (FP) program is one of the key components of public health intervention in developing countries and international development assistance programs [1]. It is defined as the ability of individuals and couples to anticipate and reach their desired number of children, through the use of contraceptive methods [2]. Long-acting reversible contraceptive methods (implants and intrauterine contraceptive device (IUCD)) are among the modern subclasses of contraceptive methods [3]. FP service is a part of maternal health services delivered to reduce maternal, infant, and child mortality in addition to enhancing the health and wellbeing of the mothers. FP services should be made available and actively promote access to all individuals desiring to decide the number and spacing of their children [2, 3].

FP is a human right and is crucial to women's empowerment, reduce poverty, raise female productivity, lower fertility, and improve child survival and maternal health. It can reduce 20–35% of maternal deaths [4, 5]. The Ethiopian Reproductive Health strategy sets the provision of all FP methods with special emphasis on long-acting reversible contraceptives (LARCs) as a key strategy of attaining its primary goals of reducing unwanted pregnancies and enabling individuals to meet their desired family size [5, 6].

The world's population growth becomes an urgent global problem, according to the 2017 revision. It is also expected that half of the world's population growth will be concentrated in just nine countries, of which Ethiopia is with a projecting population of 191 million in 2050 which makes the 10th most populous country in the world [7]. In low-income countries, the unmet need for FP leads to unintended pregnancies and in most ends up with unsafe abortion, resulting in 67,000 maternal deaths [8]. Sub-Saharan Africa countries, including Ethiopia, face reproductive health challenges, which is indicated by higher fertility, maternal, and child mortality rates. In Ethiopia, the utilization rate of LARCs is still low and dominated by the short-term contraceptive methods, which may result by gender inequalities in decisions of FP methods, religion, preferences for larger families, traditions of women marrying at an early young age, and the high drop-out rates of girls from school [9–12]. Similarly, in the Amhara region, the EDHS 2016 report shows that the utilization of LACMs is 15.1%, which is low, whereas the utilization of injectable contraceptive method is 63% with unclear reason [3, 13].

## 2. Methods

### 2.1. Study Area and Period

The study was conducted in East Gojjam Zone, Northwest Ethiopia, from 1 to 30 March 2018. The capital city is Debre Markos found 300 km far from Addis Ababa and 268 km from Bahir Dar. It has 18 districts with one referral hospital, eight district hospitals, 102 health centers, 406 health posts, six specialty clinics, 24 medium clinics, 72 primary clinics, and 58 pharmacies. There are around 2959 health care providers; of these, 1809 (61%) are females [13].

### 2.2. Study Design

We used an institutional-based cross-sectional study design.

### 2.3. Study Population

Those female health care workers work at the selected district hospitals.

### 2.4. Inclusion and Exclusion Criteria

The study included all female health care providers who are in the reproductive age group and excluded female health care workers who were recruited within 6 months during the data collection period and pregnant.

### 2.5. Sample Size Determination

The sample size was determined by using a single population proportion formula by considering the following statistical assumptions: 5% margin of error, 95% confidence interval (*α* (alpha) = 0.05), and since long-acting contraceptive methods utilization rate and the *p* value for health care workers could not be found, *p* was taken as 50%. The final sample size with a 10% adjustment for the nonresponse rate was 423. Since the difference between the calculated sample size and the total female health care workers in the selected districts was not significant, female health care workers (430) were included in the study.

### 2.6. Sampling Procedure

From the total of 18 districts in East Gojjam Zone [13], four districts (22%) were randomly selected by using the lottery method. Finally, the study participants were accessed based on the health care workers register taken from each district health office.

### 2.7. Data Collection Tools and Procedure

The questionnaire was adapted from previously done researches with some modifications to address the study objectives [14, 15]. The data were collected through a structured and self-administered questionnaire which was prepared first in English and then translated to the Amharic version.

### 2.8. Data Quality Assurance

Training was given to supervisors and the data collectors about their roles to ensure the consistency and completeness of the gathered data. A pretest was done for 21 female HCWs in Bichena town. During the data collection, the quality was monitored by the delegated supervisors and data collectors based on the daily basis performance and it was also rechecked by the principal investigator to maintain the quality of data.

### 2.9. Data Processing and Analysis

The data was entered using Epi-info Version 7 and then exported to SPSS 21 Version software for analysis. Descriptive statistics were described in tables and graphs. Binary logistic regression analysis was done to determine the preliminary relationship between the dependent and independent variables. The goodness of fit was checked using Hosmer-Lemeshow goodness of fit. Finally, factors that were found significant at *p* < 0.25 on binary logistic regression analysis were selected to perform multivariate logistic regression to identify factors significantly associated with the utilization of long-acting contraceptive methods. Results from the multivariate logistic regression were reported in the form of adjusted odds ratio at 95% CI with 0.05 levels of significance.

### 2.10. Ethical Consideration

Ethical clearance was received from the Ethical Review Committee of Debre Markos University and permission also was given from each district. Before data collection, all the study participants were informed about the purpose of the study, the risk, and benefits of the study, confidentiality of their information, and the right to quit participating in the study. To ensure confidentiality, the name of the interviewees was not written on the questionnaire.

## 3. Result

### 3.1. Sociodemographic Characteristics

A total of 392 reproductive age female health care workers were included in the analysis making the response rate 91.2%. The mean age of the participants was 27.4 years with SD ± 4.6 years. Almost all respondents were Amhara (98.7%) and Orthodox (337 (95.2%)) in ethnicity and religion, respectively. Nearly two-thirds of the participants were married (68.4%) and had a diploma educational level (65.1%). Most of the participants (88.8%) were employed in governmental health institutions; about one-third (37.2%) were nursing in their professional. Regarding income, the majority (241 (61.5%)) earned less than 5000 EBrr per month (Table 1).

### 3.2. Reproductive Health History Factors

Four out of five participants (80.1%) have started sexual intercourse with the mean age 21.31 (SD ± 3.345) years, and the majority, 281 (71.7%), of them made their first sex at the age of 18 years and above. The mean age of first birth for those who gave birth was 24.37 (SD ± 3.33) years and 62% of the participants want to have 3-4 children. 17.8% of the participants who have given birth had history of abortion; of these, 12 (35.3%) faced abortion two and more times (Table 2).

### 3.3. Attitudes towards the Utilization of Long-Acting Contraceptive Methods

Concerning the attitude of the respondents towards the use of LARCs, 48% of the female health care providers had no supportive attitude.

### 3.4. The Utilization of Long-Acting Contraceptive Methods

**Figure 1:**
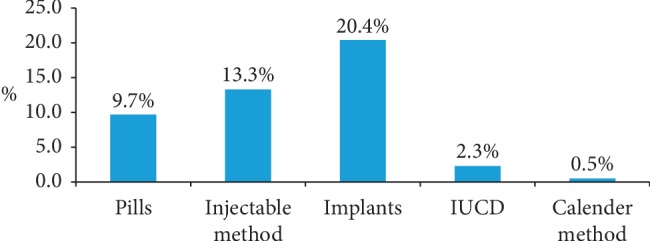
Utilization of currently contraceptive methods by method type among reproductive age female health care workers in East Gojjam Zone, Northwest Ethiopia, in 2018.

The result of this study revealed that 46.2% of the female health care workers were using any of the contraceptive methods. Nearly one out of five (20.4%) participants were using implants and only 2.3% of respondents were using intrauterine devices. Most of LARC users (86.7%) gained the service from governmental health institutions (Figure 1).

### 3.5. Factors Associated with the Utilization of Long-Acting Contraceptive Methods

To identify the factors associated with the utilization of long-acting contraceptive methods, bivariable and multivariate binary logistic regressions were used. Age group, marital status, age at first marriage, age at first birth, child want, having birth given, monthly family income, contraceptive method shift, husband feeling towards LARCs use, got training, and attitude towards LARCs utilization were entered into multivariate binary logistic regression analysis. Finally, monthly family income, having a supportive attitude, partner supportive attitude, and desired number of children have been found to be significantly associated with the utilization of long-acting contraceptive methods among health care providers.

Participants who had less than 5000 EBrr monthly family income were almost three times (AOR 2.81, 95% CI (1.04–7.57)) more likely to use long-acting contraceptive methods than those who had greater than or equal to 5000 EBrr. Female health care workers who had a supportive attitude were five times (5.13 (2.03–12.95)) more likely to use long-acting contraceptive methods than those who had a nonsupportive attitude. Those female health care service providers who had a supportive attitude from their husbands/partners towards LACMs use were also almost five times (AOR at 95% CI = 4.62 (1.52–14.09)) more likely to use LACMs than those who had neutral and refusal attitudes towards LACMs use.

Female health care service providers who want to have 0–2 children were five times (AOR at 95% CI = 5.34 (1.80–15.80)) more likely to use long-acting contraceptive methods than those who want to have 3-4 children and more (Table 3).

## 4. Discussion

This study was focused on the utilization of LARCs and associated factors among female health care service providers in the reproductive age group. In this study, the utilization of long-acting contraceptive methods was found 22.7% at 95% CI 18.4–26.5 (implant 20.4% and IUCD 2.3%). The result of this study is consistent with the studies conducted in Siltie Zone (18.4) [15], Debre Birhan university students (23.4%) [16], western Ethiopia (20%) [17], and SNNPR (26%) [18], but it is lower when compared with the studies conducted in Pakistan (76%) (implants 41% and IUCD 35%) [19], Uganda (31.7%) [20], Adama (27.9%) (implants 22% and IUCD 5.9%) [14], Gondar (34.7%) (implants 28.4% and IUCD 5.7%) [21], and Ambo Town (63.2%) (implants 57% and IUCD 6.2%) [22]. This difference might be due to the difference in the study population. This study was conducted only among female health care workers in reproductive age, but those studies were conducted among reproductive age, married women, and contraceptive users with the same study design. In general, it was observed that the utilization of LARCs is lower in the study area compared to most studies and the national target.

On the other hand, this study result on the utilization of LARCs is higher than the prevalence reported from EDHS 2016 nationally (10%) (8% implants and 2% IUCD) and Amhara region (15.1%) (12.1% Implants and 3% IUCD) [3] and Arba Minch town (13.1%) [23]. This difference might occur due to the study design and study population differences; this study was institution-based, conducted on educated and government-employed study population with highly accessed information and services. But other studies were conducted in the community-based design and in different groups of the population which means having different socioeconomic status and educational level.

Those female health care workers who had a supportive attitude from their husbands/partners towards the utilization of long-acting contraceptive methods were five times (AOR at 95% CI = 4.62 (1.52–14.09)) more likely to use long-acting contraceptive methods than those who had neutral and refusal attitudes. The result of this study is parallel to the study conducted in Adama town [14]. This may be due to the fact that Ethiopian wives including female health care service providers have values and respect the attitude of their partners/husbands.

In this study, the desired number of children has shown an association with the utilization of long-acting contraceptive methods. Female health care workers who want to have 0–2 children were five times (AOR 5.34 95% CI (1.80–15.80)) more likely to use long-acting contraceptive methods than those who want to have 3-4 children and more. This finding was supported by research done on the determinants of the utilization of LARCs using evidence from 2011 [24]. This may be due to the fact that women who want to stop or extend fertility may choose effective long-acting contraceptive methods from the available options.

The result of this study revealed that those who had less than 5000 EBrr monthly family income were three times (AOR at 95% CI = 2.813 (1.04–7.57)) more likely to use long-acting contraceptive methods. This might be related to the fact that families with lower monthly income hardly afford the cost of rearing children; hence, they might be enforced to use long-acting contraceptive methods for effective and durable prevention of pregnancy because they have better knowledge and skills on LARCs. But a study conducted in Ambo town shows the opposite of this study result; it showed that “those mothers who had income greater than five hundred birrs per month were 4 times more likely to use LARCs as compared with those who had low income. This was reasoned as mothers who have good income can learn and can get information from media easily” [22].

The other significant factor which affects the utilization of long-acting contraceptive methods is the attitude of female health care workers towards the utilization of long-acting contraceptive methods; those who had supportive attitude were five times (5.13 (2.03–12.95)) more likely to use long-acting contraceptive methods than those who had a nonsupportive attitude. The result is supported by the findings from Siltie Zone [15], Arba Minch town [23],and Ambo Town [22]. Women who had supportive attitudes towards long-acting contraception are more likely to use long-acting contraceptive methods than those who had nonsupportive attitudes. This might be due to having a positive attitude that helped them to resist misconception and tolerate the side effects of using long-acting contraceptive methods.

In studies conducted in Adama discussion with partners/husbands, ever use of long-acting contraceptive methods and governmental employed women were positively associated with the utilization of long-acting contraceptive methods [14]. The other study conducted in Siltie Zone [15] showed that having secondary and above educational status, high knowledge level on LARCs, and positive attitude on LARCs were affected positively by the increasing utilization of long-acting contraceptive methods. These factors might not affect the utilization of LARCs among female health care service providers due to the similarities in educational level and occupational status of the study units.

## 5. Conclusion

The study revealed the low utilization of long-acting contraceptive methods among female health workers. There was also a difference in the utilization of the specific methods of long-acting contraceptive methods with very low utilization of the IUCD method. Husbands/partners' attitude towards the utilization of LARCs, the desired number of children, the attitude towards the utilization of LARCs, and monthly family income were statistically significant factors with the utilization of LARCs.

## Figures and Tables

**Table 1:** Sociodemographic characteristics of the utilization of long-acting contraceptive methods among reproductive aged female health care workers in East Gojjam Zone, Northwest Ethiopia, in 2018.

Sociodemographic variables of the respondent	Frequency	Percent (%)
Age group (*N* = 392)		
24 or less	105	26.8
25 or more	287	73.2
Marital status (*N* = 392)		
Single	124	31.6
Married	268	68.4
Educational level (*N* = 392)		
Certificate	36	9.2
Diploma	255	65.1
Degree and above	101	25.8
Current place of work (*N* = 392)		
Governmental health institution	348	88.8
Private health institution	36	9.2
NGO health institution	8	2.0
Current work position/profession (*N* = 392)		
Nurse	146	37.2
Health extension worker	86	21.9
Midwifery	39	9.9
Pharmacy	47	12.0
Health officer	27	6.9
Laboratory	24	6.1
HIT^*∗*^	15	3.8
Others^*∗∗*^	8	2.0
Husband's/friend's educational level (*N* = 296)		
Secondary school and lower class	33	8.4
Certificate and above	263	67.1
Husband's/friend's occupational status (*N* = 296)		
Government employee	241	61.5
Merchant	32	8.2
Others^*∗∗∗*^	23	5.9
Monthly family income in EBrr (*N* = 392)		
<5000	242	61.7
≥5000	150	38.3

^*∗*^Health information technician. ^*∗∗*^Anesthetist, environmentalist, and radiologist. ^*∗∗∗*^Derivers and handcraft maker.

**Table 2:** Reproductive health history of the utilization of long-acting contraceptive methods among reproductive aged female health care workers in East Gojjam Zone, Northwest Ethiopia, in 2018.

Reproductive history of study participants	Frequency	Percent (%)
Started sexual intercourse (*N* = 392)		
No	78	19.9
Yes	314	80.1
Age at first sex (*N* = 314)		
<18 years	33	8.4
≥18 years	281	71.7
Age at first marriage (*N* = 288)		
<18 years	35	8.9
≥18 years	253	64.5
Ever given birth (*N* = 314)		
No	123	31.4
Yes	191	48.7
Age at first birth (*N* = 191)		
<18 years	5	1.3
≥18 years	186	47.4
Number of births given (*N* = 191)		
1-2 times	156	39.8
3-4 times	34	8.7
≥5 times	1	0.3
Number of having alive children (*N* = 191)		
1-2 children	154	39.3
3-4 children	34	8.7
≥5 children	1	0.3
Number of children they want to have (=392)		
0–2 children	87	22.2
3-4 children	243	62.0
≥5 children	62	15.8
Had abortion history (*N* = 392)		
No	358	91.3
Yes	34	8.7
Number of abortion experienced (*N* = 34)		
1 time	22	5.6
≥2 times	12	3.1

**Table 3:** Factors associated with the utilization of long-acting contraceptive methods among reproductive aged female health care workers in East Gojjam Zone, Northwest Ethiopia, in 2018.

Independent variables	Utilization of LARC		COR at 95% CI	AOR at 95% CI
	Yes	No		
Marital status				
Single	8 (9%)	116 (38.3%)	1	1
Married	81 (91%)	187 (61.7%)	6.28 (2.93–13.46)	3.73 (0.39–36.15)
Method shift				
Yes	52 (58.4%)	83 (44.4%)	1	1
No	37 (41.6%)	104 (55.6%)	0.57 (0.34–0.95)	1.42 (0.45–4.49)
Method shift for new method				
Yes	31 (34.8%)	43 (14.2%)	1	1
No	58 (65.2%)	260 (85.8%)	0.31 (0.18–0.53)	0.54 (0.22–1.33)
Method shift due to side effects				
Yes	23 (25.8%)	31 (10.2%)	1	1
No	66 (74.2%)	272 (89.8%)	0.33 (0.19–0.60)	0.73 (0.20–2.58)
Health care workers counseling				
Yes	10 (11.2%)	11 (3.6%)	1	1
No	79 (88.8%)	292 (96.4%)	2.98 (0.12–0.73)	0.64 (0.12–3.51)
Got special training				
Yes	79 (27.1%)	213 (72.9%)	3.34 (1.65–6.74)	0.56 (0.16–2.02)
No	10 (11.2%)	90 (29.7%)	1	1
Attend on-the-job training				
Yes	70 (78.7%)	56.1 (%)	2.89 (1.65–50)	1.66 (0.26–10.47)
No	19 (21.3%)	133 (43.9%)	1	1
Age at first marriage				
<18 years	16 (18.8%)	19 (9.4%)	1	1
≥18 years	69 (81.2%)	182 (990.6%)	0.45 (0.22–0.92)	0.36 (0.08–1.70)
Age at first birth given				
<18 years	4 (6.7%)	1 (0.8%)	1	1
≥18 years	56 (93.3%)	130 (99.2%)	0.11 (0.01–0.99)	0.00
Age group				
<24 years	14 (15.7%)	91 (30.0%)	0.44 (0.23–0.81)	0.34 (0.06–2.05)
≥24 years	75 (84.3%)	212 (70.0%)	1	1
Monthly family income				
<5000	70 (78.7%)	171 (56.6%)	2.82 (1.61–4.92)	2.81 (1.04–7.57)^*∗*^
≥5000	19 (78.7%)	131 (43.4%)	1	1
Number of children they want to have				
0–2 children	56 (62.9%)	31 (10.2%)	14.89 (8.43–26.29)	5.34 (1.80–15.80)^*∗*^
≥3 children	33 (37.1%)	272 (70.0%)	1	1
Husband/partner's feeling towards using LARCs				
Supportive	79 (88.8%)	79 (43.6%)	10.2 (4.96–20.96)	4.62 (1.52–14.09)^*∗*^
Neutral and refusal	10 (9%)	102 (56.4%)	1	1
Attitude towards utilization of LARCs				
Supportive	72 (80.9%)	116 (38.3%)	6.83 (3.83–12.16)	5.13 (2.03–12.95)^*∗*^
Nonsupportive	17 (19.1%)	187 (61.7%)	1	1

^*∗*^Statistically significant (*p* value <0.05).

## Data Availability

The data used to support the findings of this study are available from the corresponding author upon request.
