# Corrigendum to “Utilization of Long-Acting Contraceptive Methods and Associated Factors among Female Health Care Providers in East Gojjam Zone, Northwest Ethiopia, in 2018”

**DOI:** 10.1155/2020/2965314

**Published:** 2020-09-08

**Authors:** Manaye Meku Gella, Liknaw Bewket Zeleke, Hunegnaw Almaw Derseh, Addisu Alehegn Alemu, Eskeziaw Abebe Kassahun, Kelemu Abebe Gelaw

**Affiliations:** ^1^Debremarkos Town Health Office, Bahir Dar, Ethiopia; ^2^College of Health Sciences, Debre Markos University, Debre Markos, Ethiopia; ^3^College of Health Sciences and Medicine, Bahir Dar University, Bahir Dar, Ethiopia; ^4^Faculty of Health Sciences, Woldia University, Woldia, Ethiopia; ^5^College of Health Sciences and Medicine, Wolaita Sodo University, Wolaita Sodo, Ethiopia

In the article titled “Utilization of Long-Acting Contraceptive Methods and Associated Factors among Female Health Care Providers in East Gojjam Zone, Northwest Ethiopia, in 2018” [1], there was an error in the author order, where first authorship should have been assigned to Manaye Meku Gella.

The correct author order is shown below and also corrected in the author list:

Manaye Meku Gella, Liknaw Bewket Zeleke, Hunegnaw Almaw Derseh, Addisu Alehegn Alemu, Eskeziaw Abebe Kassahun, and Kelemu Abebe Gelaw.

## References

[1] Zeleke L. B., Gella M. M., Derseh H. A., Alemu A. A., Kassahun E. A., Gelaw K. A. (2019). Utilization of long-acting contraceptive methods and associated factors among female health care providers in East Gojjam Zone, Northwest Ethiopia, in 2018. *BioMed Research International*.

